# Clinical and Radiological Outcomes Comparison of Degradable Starch Microspheres TACE with Idarubicin vs. Epirubicin Protocol in Patients with HCC

**DOI:** 10.3390/diagnostics16071100

**Published:** 2026-04-05

**Authors:** Francesco Giurazza, Pietro Roccatagliata, Claudio Carrubba, Fabio Corvino, Raffaella Tortora, Marco Guarracino, Mariafiorella Brangi, Carla Migliaccio, Federica Falaschi, Maria Cammarota, Raffaella Niola

**Affiliations:** 1 Vascular and Interventional Radiology Department, Cardarelli Hospital, Via Antonio Cardarelli 9, 80131 Naples, Italy; pietro.roccatagliata@aocardarelli.it (P.R.); claudio.carrubba@aocardarelli.it (C.C.); fabio.corvino@aocardarelli.it (F.C.); raffaella.niola@aocardarelli.it (R.N.); 2 Hepatology Department, Cardarelli Hospital, Via Antonio Cardarelli 9, 80131 Naples, Italy; raffaella.tortora@aocardarelli.it (R.T.); marco.guarracino@aocardarelli.it (M.G.); 3 Oncology Department, Cardarelli Hospital, Via Antonio Cardarelli 9, 80131 Naples, Italy; mariafiorella.brangi@aocardarelli.it; 4 Hepatobiliary Surgery Department, Cardarelli Hospital, Via Antonio Cardarelli 9, 80131 Naples, Italy; carla.migliaccio@aocardarelli.it (C.M.); federica.falaschi@aocardarelli.it (F.F.); 5 Pharmacy Department, Cardarelli Hospital, Via Antonio Cardarelli 9, 80131 Naples, Italy; maria.cammarota@aocardarelli.it

**Keywords:** computed tomography, clinical, HCC, TACE, degradable starch microspheres (DSM), epirubicin, idarubicin

## Abstract

**Background/Objectives**: Transarterial chemoembolization (TACE) is included in international guidelines for the treatment of hepatocellular carcinoma (HCC), but it is still not a standardized intervention in terms of vector and chemotherapy. This study aims to report on clinical and radiological outcomes of degradable starch microspheres TACE (DSM-TACE) with idarubicin and compare with DSM-TACE with an epirubicin protocol after a single session. **Methods**: This is a single-center retrospective study analyzing cirrhotic patients affected by HCC in early or intermediate stages. Primary objectives were to assess the safety and efficacy of a single DSM-TACE with 10 mg idarubicin in terms of adverse event (AE) occurrences evaluated via the CTCAE 5.0 system and mRECIST criteria with computed tomography (CT) at 3 months. The secondary purpose was to compare the procedural outcomes with those from patients treated with DSM-TACE with 50 mg epirubicin. **Results**: Thirty-seven patients were included, 19 treated with idarubicin (IDA group) and 18 with epirubicin (EPI group); demographic data and lesion characteristics were comparable. No major AE (grade ≥ 3) occurred overall. In the IDA group, the minor AE incidence was 52.7%: one patient presented with mild ascites, eight developed hyperbilirubinemia and one leucopenia. At the 3-month CT follow-up, mRECIST criteria reported an overall response rate (ORR) of 63.2% and a disease control rate (DCR) of 84.2%. No statistically significant differences were appreciable comparing both AE occurrence and mRECIST findings with the EPI group (50% minor AE, 77.8% ORR, 88.9% DCR). **Conclusions**: In this sample of cirrhotic patients with HCC, DSM-TACE with 10 mg idarubicin was safe and effective comparable to DSM-TACE with 50 mg epirubicin.

## 1. Introduction

Hepatocarcinoma (HCC) accounts for approximately 90% of liver cancer, which in turn represents the third cause of cancer-related death worldwide [1]. The majority of patients are not suitable for curative treatments such as resection, ablation or transplantation at the time of diagnosis, so palliative intent is applied in many cases [2].

In patients not eligible for curative strategies, transarterial chemoembolization (TACE) is one of the most commonly selected options. TACE has been included in the international guidelines for decades [3], especially by the Barcelona Clinic Liver Cancer (BCLC) for early and intermediate stages [1,4], with the latter proposing it as a first-line therapy.

Nevertheless, from a technical standpoint, TACE is not a standardized intervention as there is no proper consensus in terms of ideal carrier or chemotherapy.

There are three main different TACE techniques to be considered, namely cTACE (conventional TACE, performed with a drug-Lipiodol^®^ emulsion), DEB-TACE (performed with drug-eluting beads) and more recently DSM-TACE (done with degradable starch microspheres); the most significant distinction between the more commonly used DEB-TACE and cTACE is the short-lived and well-defined transient vascular occlusion that characterizes DSM-TACE. Despite the extensive literature on TACE in HCC, there is no scientific evidence supporting the superiority of one technique over another, and the choice is reported to be mainly operator-dependent [5].

A similar variability of options is applicable to the different chemotherapies available with the types of TACE. The most commonly adopted drugs in clinical practice for HCC are the anthracyclines doxorubicin and epirubicin (a stereoisomer of doxorubicin), at an average dosage of 50 mg [6,7], even if these present limited efficacy, with a response rate only up to 60% [8,9].

Surprisingly, this choice is not supported by any level I scientific data in the absence of randomized controlled trials.

In 2011 a French study performed in vitro testing to compare the efficacy of eleven chemotherapeutical drugs on three human HCC cell lines [10]. Results showed a net superiority of idarubicin, which is an anthracycline mainly adopted in hematological malignancies. Since then, different studies on humans have supported the role of idarubicin in both cTACE [11] and DEB-TACE [12,13,14,15,16] for the treatment of HCC, but today no data are available on the use of idarubicin with DSM-TACE.

For this reason, this study aims to fill this gap. It is the first one to report clinical and radiological outcomes of DSM-TACE with idarubicin and to compare results with DSM-TACE with epirubicin after a single treatment session.

## 2. Materials and Methods

### 2.1. Study Design

This is a single-center retrospective study analyzing all consecutive patients treated with DSM-TACE during 2025.

This study received approval from the local ethical committee (DEL 233/2024), and all patients signed a written informed consent to receive the intervention and participate in this study.

Inclusion criteria were: patients affected by HCC based on EASL criteria [1], BCLC stages A (early) or B (intermediate), underlying cirrhosis, DSM-TACE, and follow-up available up to 3 months.

Exclusion criteria were: ruptured HCC, previous liver-directed endovascular therapies (cTACE/DEB-TACE or radioembolization), portal vein occlusion of segmental or larger branches (>VP 2) [17], absolute contraindications to endovascular therapies (coagulation impairment, renal failure), and intolerance to chemotherapy with epirubicin or idarubicin.

The local electronic dataset was reviewed to retrieve the following data: demographic information, laboratory values (blood count, bilirubin, transaminases, albumin, coagulation status), diagnostic and interventional imaging, procedural reports, and clinical evaluations.

The primary objectives of this study were safety and efficacy evaluations of a single DSM-TACE with idarubicin in terms of adverse event (AE) occurrences, overall response rate (ORR) and disease control rate (DCR). The secondary aim was to compare the procedural outcomes of DSM-TACE with idarubicin with DSM-TACE with epirubicin.

### 2.2. TACE Interventions

All patients were treated in an in-hospital setting with a one-night stay in either the hepatology, oncology or liver surgery department.

The indication for TACE was the result of a multidisciplinary team evaluation, performed by hepatologists, oncologists, liver surgeons, nuclear physicians, and diagnostic and interventional radiologists. In this series both palliative (disease control) and bridge to transplant TACE procedures were considered. The choice of DSM-TACE in particular was made by the interventional radiologist, according to the patient profile characteristics, and all operators involved followed a standard local protocol. The drug combined with the starch microspheres was not randomly assigned; since June 2025, due to a local shortage of epirubicin, only idarubicin became available.

Liver function was checked in all patients before the intervention adopting the CP (Child–Pugh) classification; in all cases, CP was A or <B7.

With regards to the procedural set up, contrast-enhanced computed tomography (CT) or magnetic resonance (MR) performed at most 30 days before the intervention was studied in order to select an appropriate treatment strategy in terms of vascular anatomy and lesion targeting.

Laboratory data, including blood count, liver and renal function, as well as an electrocardiogram (ECG) were evaluated during an anesthesiologic consultation before each TACE to assess procedural feasibility.

**Figure 1:**
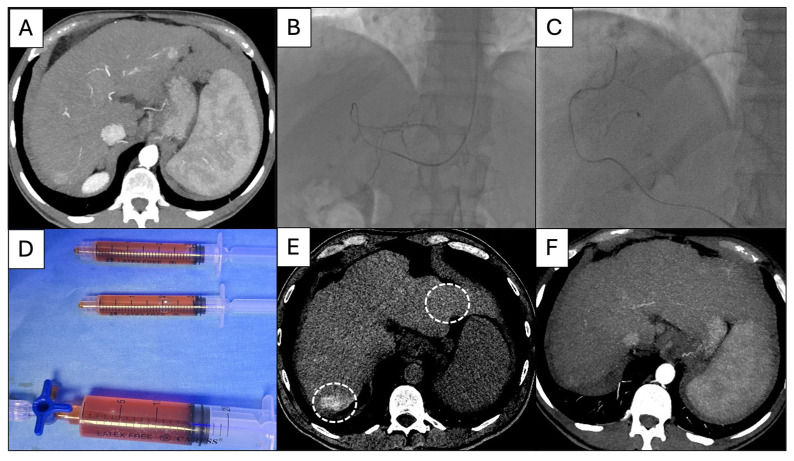
DSM-TACE with idarubicin performed according to a standard protocol. A 75-year-old male affected by HCV-related cirrhosis and HCC BCLC B. (**A**) In CT, 3 hypervascularized HCC nodules in arterial phase were evident in S7, S2 and S5. (**B**,**C**) Via right radial access, a 5 Fr diagnostic catheter (headhunter or multipurpose tip) was advanced in the common hepatic artery and then a 2.4 Fr microcatheter was coaxially positioned superselectively to the nodule feeders. (**D**) Here 4 mL of DSM was mixed with idarubicin (10 mg Zavedos^®^ diluted in 7 mL of saline) and 9 mL of pure iodinated contrast agent, then injected via 3 mL Luer lock syringes; after chemotherapy, temporary embolization was obtained with 3.5 mL of DSM mixed with 6.5 mL of pure iodinated contrast agent. (**E**) Plane CT scan acquired in the angio suite immediately after TACE, depicting nodule opacification (white dotted circles). (**F**) The 3-month follow-up CT in arterial phase revealing net enhancement reduction in the nodule in S7 and disappearance of the nodule in S2, compatible with PR at mRECIST criteria evaluation.

Endovascular interventions (Figure 1) were performed across two angio suites equipped with modern generation C-arms integrated by ConeBeamCT (CBCT) (Axiom Artis Zee, Siemens^®^, Erlangen, Germany) or CT (Alphenix, Canon^®^, Tokyo, Japan). All interventional radiologists had more than 10 years of experience in liver-directed interventions.

Arterial access was performed under ultrasound (US) guidance by positioning a 5 Fr introducer in the right radial artery; in the case of anatomical complexity, the right femoral artery was accessed instead. Following puncture, the coeliac trunk and superior mesenteric artery were selected with a 5 Fr diagnostic catheter to acquire a complete arterial mapping of the liver and identify all HCC nodules.

After diagnostic angiography, a superselective catheterization was performed through a 2.4 Fr microcatheter, which was placed in the right and/or left hepatic artery according to the lobe involved, then distally advanced into the target nodule feeders (Figure 1B,C). Selective angiography was performed to confirm the correct positioning of the microcatheter, identify non-hepatic arteries and limit potential non-target embolization. Contrast-enhanced CBCT or CT were acquired to identify proper nodule targeting before particle and drug administration.

Under fluoroscopic guidance, a solution of DSM (Embocept S, Magle PharmaCept, Berlin, Germany) mixed with either injectable lyophilized epirubicin (Farmorubicin, Pfizer^®^, New York, NY, USA) or idarubicin (Zavedos, Pfizer^®^, New York, NY, USA) and contrast medium, followed by pure Embocept mixed with contrast medium, was slowly and continuously infused. Embocept S microspheres are degradable starch particles presenting a mean diameter of 50 μm (range: 20–90 μm) and a half-life in vivo of 40 min, with complete reabsorption in 100 min, mediated by α-amylase enzymes.

In detail, a “two-step” infusion technique was used [18] as follows: A “drug uptake” phase in which the DSM + drug solution (4 mL of microspheres + 50 mg epirubicin or 10 mg idarubicin, both diluted in 7 mL saline solution, +9 mL contrast medium) was slowly intra-arterially injected; the injection technique consisted of intermittent tapping on the 3 mL syringe piston (Figure 1D) to allow particle distal penetration and minimize reflux; then, a “stopped-flow” phase in which DSM (3.5 mL) mixed with contrast medium (6.5 mL) was slowly injected to temporary occlude the main hepatic branches according to the nodules position. The procedural endpoint was complete therapy delivery or flow stasis in the feeding artery, lasting for 5 beats per minute.

Plane CBCT or CT was performed to confirm technical success defined by nodule opacification (Figure 1E).

A compressive wrist bracelet or a mechanical closure device (Angioseal or Femoseal, Terumo^®^, Tokyo, Japan) was positioned at the end of each intervention in case of radial or femoral access, respectively.

The following anesthesiologic support was available for all procedures: 30 min before entering the angio suite, patients were administered an intramuscular opioid (meperidine) together with endovenous corticosteroids (dexamethasone), an antiemetic (ondansetron) and gastric protection (pantoprazole); local anesthesia was injected before positioning the 5 Fr introducer; endovenous light sedation (midazolam) was administered immediately before particle injection.

After TACE, 1 gr of endovenous paracetamol diluted in 100 mL of saline was given to all patients, who were required to fast for 5 h.

### 2.3. Follow-Up and Outcome Evaluation

TACE effects were monitored with clinical, laboratory and radiological examinations.

A clinical evaluation was conducted by the leading physician of the hospitalization department during the stay and monthly thereafter.

Laboratory data included blood count, liver function and coagulation status and were evaluated up to 30 days after treatment.

Safety was evaluated according to adverse event occurrences, applying the CTCAE 5.0 grading system [19].

A contrast-enhanced CT scan was acquired within three months to assess tumor response and determine the need for additional TACE.

The radiological CT scan analysis was performed blindly to the treatment type and clinical data; TACE response efficacy was assessed according to mRECIST criteria [20] through complete response (CR), partial response (PR), stable disease (SD) and progressive disease (PD).

### 2.4. Statistical Analysis

Descriptive statistics are reported as mean ± SD for normally distributed continuous variables and as median (IQR) otherwise; categorical variables are reported as *n* (%). Normality was assessed using the Shapiro–Wilk test. Between-group comparisons (idarubicin vs. epirubicin) were performed using Student’s *t* test (or Welch’s *t* test when appropriate) for normally distributed continuous variables and the Mann–Whitney U test otherwise; categorical variables were compared using Fisher’s exact test (or Chi-square test when appropriate). Peak post-TACE laboratory values were defined as the maximum value recorded within 30 days after the procedure. Analyses were conducted on available cases for each variable; missing data were not computed. All tests were two-sided with *p* < 0.05 considered statistically significant. Analyses were performed using Microsoft Excel (version 2024, Microsoft^®^ Corp., Redmond, WA, USA) and Python (version 3.14.3, Python Software Foundation; NumPy/SciPy) software.

## 3. Results

### 3.1. Sample Description

**Figure 2:**
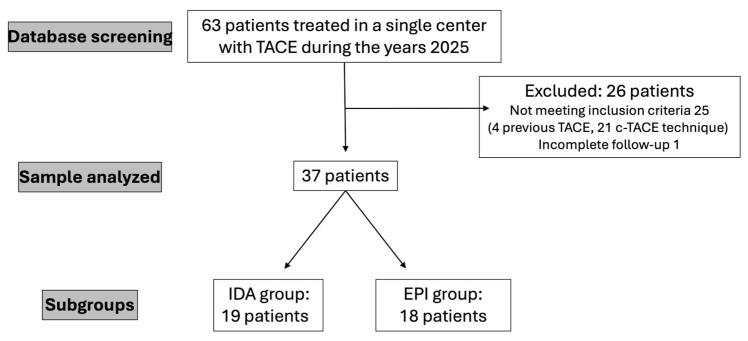
CONSORT flowchart describing study population selection.

Sixty-three patients were treated with TACE and 37 met the inclusion criteria (Figure 2), receiving 37 DSM-TACE.

Nineteen patients were treated with idarubicin (IDA group) and 18 with epirubicin (EPI group).

Demographic data and lesions characteristics were comparable (*p*-value > 0.05) among the two groups (Table 1): mean age was 65 years (range: 57.5–76) with 17 males and 2 females in the IDA group and 70 years (range: 63.2–82.7) with 15 males and 3 females in the EPI group.

In the IDA group, the mean maximum HCC diameter was 31 mm (range: 24–43), with 7 patients (36.8%) in BCLC A and 12 patients (63.2%) in BCLC B; in the EPI group the mean maximum HCC diameter was 28.5 mm (range: 20.2–42.2), with 8 patients (44.5%) in BCLC A and 10 (55.5%) in BCLC B.

Cirrhosis etiologies in the IDA group were: infective (HCV or HBV or combo) in 10 patients (52.6%), cryptogenic in 3 (15.8%), dysmetabolic in 3 (15.8%), alcohol in 2 (10.5%), combination of HCV and alcohol in 1 (5.3%); in the EPI group they were infective (HCV or HBV or combo) in 9 patients (50%), cryptogenic in 3 (16.7%), dysmetabolic in 3 (16.7%), alcohol in 2 (11.1%), combination of HCV and alcohol in 1 (5.5%).

Regarding previous treatments, in the IDA group, four patients (21%) had been treated with surgical resections and eight (42.1%) with ablation, while in the EPI group, three patients (16.7%) had been treated with surgical resections and five (27.8%) with ablation.

### 3.2. Safety and Efficacy Analysis IDA Group

After one TACE session, no major adverse events (AE grade ≥ 3) occurred. One patient (5.3%) presented with mild ascites 1 week after TACE (AE grade 2) and was treated with diuretic therapy at home. No episodes of fever, vomiting and abdominal pain were recorded.

Regarding laboratory findings, nine patients (47.4%) developed asymptomatic AE grade 1 events: eight patients experienced a transient increase in bilirubin levels within 24 h from TACE, which normalized by one week, and one patient had transient leucopenia. No anomalies in transaminases, albumin and coagulation values were detected.

At the 3-month CT follow-up, the mRECIST evaluation showed CR in three patients (15.8%), PR in nine patients (47.4%), SD in four subjects (21%), and PD in three (15.8%); ORR was 63.2% and DCR was 84.2%.

A second TACE session was rescheduled for those patients with SD and PD, according to clinical laboratory conditions.

### 3.3. Comparison with EPI Group

*Safety and efficacy analysis EPI group*

No major adverse events occurred in the EPI group. Two patients (11.1%) suffered clinical minor events (AE grade 2) in the afternoon following TACE: one patient had vomiting and fever, while a second patient had abdominal pain. Both cases were managed with medical therapy and did not require prolonged hospital stays.

Six patients (33.3%) had minor AE grade 1 in terms of clinically asymptomatic laboratory values: five patients exhibited a transient increase in bilirubin levels, and one had transient leucopenia, all normalized within 10 days and managed by hydration, with no additional medical therapy required.

At the 3-month CT follow-up, mRECIST showed CR in 4 patients (22.2%), PR in 10 patients (55.6%), SD in 2 subjects (11.1%), and PD in the remaining 2 patients (11.1%); ORR was 77.8%, and DCR 88.9%.

*Comparison between IDA group and EPI group*

No significant (*p*-value > 0.05) differences emerged when comparing both adverse event occurrences and mRECIST findings among the two groups (Table 2). No major AEs were recorded overall, and the minor AE incidence was comparable (52.7% in the IDA group vs. 50% in the EPI group, *p*-value > 0.05) between the two groups.

Furthermore, the EPI group performed better in terms of ORR and DCR compared with the IDA group (77.8% vs. 63.2% and 88.9% vs. 84.2%), but the differences were not statistically significant (*p*-value > 0.05).

## 4. Discussion

The role of TACE has been the subject of extensive investigation, with robust scientific evidence proving its safety and efficacy; however, although it has been established for decades, TACE still cannot rely on fully standardized protocols. Nevertheless, in line with the tailored approach of modern medicine, the existence of different TACE techniques does not imply a preference for one over the other; instead, it can offer a broader range of potential techniques that can be chosen to fight HCC. The success of this approach relies on the expertise of multidisciplinary oncology teams and a thorough understanding of the latest interventional possibilities.

To date there is still no consensus about the superiority of different TACE techniques nor the drug to use; yet, it is unanimously agreed upon that limited toxicity is one of the key conditions to respect in order to maintain good performance status and compensated liver disease. TACE with DSM allows the temporary occlusion of the smaller arterial tumoral vessels thanks to the short half-life and the small size of the particles; this improves the overall therapeutic efficacy by reducing the immediate wash-out of the drug and at the same time decreases the risk of systemic toxicity and post-embolic syndrome [21].

In this study, we aimed to assess the efficacy and safety profile of DSM-TACE with idarubicin, in addition to comparing these results with DSM-TACE with epirubicin.

In this sample, superselective DSM-TACE with 10 mg of idarubicin was found to be safe, without major AE occurrences, and effective, with 84.2% DCR and 63.2% ORR, at the 3-month follow-up after a single treatment session. Also, when comparing outcomes with the more established DSM-TACE with 50 mg of epirubicin, no statistically significant differences were found in terms of AE, ORR or DCR.

These findings suggest that idarubicin, largely adopted in clinical practice in hematological disorders, is a chemotherapy that could be considered with DSM-TACE as a valuable alternative to epirubicin.

From a biochemical standpoint, idarubicin is more cytotoxic compared with the other anthracyclines for three reasons: it is highly lipophilic and so has an increased penetration through the lipophilic double layer of the tumor cell membrane [10,22]; it has a sustained half-life thanks to idarubicinol, its active metabolite [23,24]; it is less affected by chemoresistance mechanisms (typical of HCC cells, as a P-glycoprotein-mediated efflux pump) with a consequent increased intracellular drug concentration [13,24].

Both in clinical practice and in the literature, TACE procedures are heterogeneous, without a clear superiority of one technique over the other. For decades the choice of doxorubicin and epirubicin has been driven more by availability and toxicity profiles than by robust comparative data; indeed, epirubicin anti-HCC efficacy is modest [25] and no randomized trials have yet proven that doxorubicin improves overall survival [26].

On the other hand, interest in idarubicin has risen in the last years, especially after an in vitro study published in 2011 showing net superior cytotoxicity on HCC cells of idarubicin, over 11 other anthracyclines, including doxorubicin and epirubicin [10].

Since then, different studies have been conducted regarding TACE with idarubicin in HCC. The first pilot one in 2012 analyzed 21 patients treated with conventional Lipiodol^®^ TACE with 10 mg idarubicin, showing a high safety profile with encouraging oncological results [11]. Two trials conducted in France, IDASPHERE phase 1 trial and IDASPHERE II phase 2 trial, focused instead on DEB-TACE using 300–500 μm particles. In IDASPHERE [12], the maximum idarubicin-tolerated dose was investigated in 21 patients, by escalating it from 5, 10 to 15 mg, concluding that the maximum tolerated dose per TACE session was 10 mg and finding a 52% ORR at 2 months. In IDASPHERE II [13], 44 patients with unresectable HCC were treated with 10mg idarubicin DEB-TACE, reporting 52% ORR at 6 months with an overall survival of 18,6 months, 9% treatment discontinuation for toxicity and main grade 3–4 AE consisting of transaminases elevation, hyperbilirubinemia and pain.

More recently, two studies compared TACE with idarubicin versus doxorubicin or epirubicin. In 2020 Roth et al. [14] compared 60 patients treated with doxorubicin and 30 with idarubicin, in the intermediate stage and mainly cirrhotic. They included both cTACE and DEB-TACE, obtaining comparable results in terms of efficacy and safety, respectively, in the doxorubicin and idarubicin groups. ORR within 3 months after first TACE was 76.7% and 73.3%, while AE grades 3–4 occurred in 35% and 43%. In 2025 Liu et al. [16] published a randomized phase IV trial including 239 patients in the intermediate stage treated with DEB-TACE with idarubicin or epirubicin. They reported significant differences in progression-free survival (10.8 vs. 8.7 months) and ORR (70.8% vs. 57.1%), in favor of idarubicin, without differences in AE incidence.

Our data represent the first-time use of idarubicin combined with DSM and can be useful in adding another drug to the list of those reported in the literature with these degradable microspheres. As already mentioned, the main advantage of DSM-TACE is the increased patient tolerability without significant differences in efficacy compared with cTACE and DEB-TACE [21,27,28]. In this study population, the low rate of AE, mainly grade 1, is in line with the previous literature data [21,27,28,29,30], confirming the high safety and tolerability of DSM-TACE, which actually has already been safely applied also in patients in the advanced stage (BCLC-C) [29,30] (related to the short reabsorption time of the microparticles). Importantly, procedural efficacy in terms of ORR was also consistent and similar to studies adopting idarubicin in cTACE and DEB-TACE. Furthermore, there is recent clinical evidence supporting the efficacy of DSM-TACE in inducing tumor necrosis rates comparable to those of DEB-TACE and cTACE despite its short embolization time, weakening the belief that prolonged embolization is necessary for achieving substantial tumor necrosis in TACE [31].

As previously described, no significant differences emerged comparing idarubicin with epirubicin in terms of AE incidence and ORR. This suggests that DSM-TACE with idarubicin is a valuable alternative to the more commonly adopted epirubicin. In addition, real-life challenges such as a recurring shortage of one or another drug may create operational blocks or delays, so having an alternative becomes the utmost importance in everyday clinical practice. Furthermore, chemotherapies different to anthracyclines, as cisplatin, can be adopted in TACE and even bland transarterial embolization can be considered as a valuable alternative [1].

Although our study shows that the use of DSM can be extended to idarubicin, it does present several limitations. First of all, the single-center retrospective design reduces the reproducibility of these findings. Secondly, the non-randomized selection of anthracycline adds experimental bias, since the choice of idarubicin was due to the local shortage of epirubicin rather than randomly assigned. This could have influenced the interventional radiologists, even if the mRECIST evaluation was blinded. However, it should also be noted that randomization would not have been technically easy due to the different color of the drugs (dark red epirubicin vs. light orange for idarubicin). Then, follow-up was conducted only up to 3 months, so long-term outcomes still have to be investigated. Finally, even if this is the first study proving efficacy and safety with idarubicin DSM-TACE, the number of patients included was still limited, and further studies with larger samples are needed to consolidate results.

## 5. Conclusions

In conclusion, in this study, DSM-TACE with idarubicin was safe and effective in a group of BCLC A- and B-stage cirrhotic patients with HCC, with radiological and clinical outcomes comparable to DSM-TACE with epirubicin. However, further prospective studies with larger patient populations and randomized design are needed to clarify the role of different types of drugs in TACE for HCC and before advocating for drug interchangeability with DSM-TACE in particular.

## Figures and Tables

**Table 1:** Patient characteristics.

Parameter	IDA Group	EPI Group	*p*-Value
Patient number	19	18	-
Mean age (range)	65 (57.5–76.0)	70 (63.2–82.7)	0.06
Sex *M* *F*	17 2	15 3	0.66
Mean max HCC size (mm) (range)	31 (24–43)	28.5 (20.2–42.2)	0.73
BCLC stage *A* *B*	7 (36.8%) 12 (63.2%)	8 (45.5%) 10 (55.5%)	1
Cirrhosis etiology *Infective* *Cryptogenic* *Dysmetabolic* *Alcohol* *Multifactor*	10 (52.6%) 3 (15.8%) 3 (15.8%) 2 (10.5%) 1 (5.3%)	9 (50%) 3 (16.7%) 3 (16.7%) 2 (11.1%) 1 (5.5%)	0.82
Previous surgery	4/19 (21%)	3/18 (16.7%)	1
Previous ablation	8/19 (42.1%)	5/18 (27.8%)	0.49

M: male; F: female; HCC: hepatocellular carcinoma; BCLC: Barcelona Clinic Liver Cancer.

**Table 2:** Adverse event occurrences and mRECIST evaluation in IDA group and EPI group.

Parameter	IDA Group (*n* = 19)	EPI Group (*n* = 18)	*p*-Value
**Safety (post-TACE adverse events, CTCAE grading)**			
Overall minor adverse events (CTCAE grade ≤ 2)	10/19 (52.6%)	9/18 (50%)	1
Clinical minor events (CTCAE grade ≤ 2)	1/19 (5.3%)	2/18 (11.1%)	0.6
Laboratory minor events (CTCAE grade 1)	9/19 (47.4%)	6/18 (33.3%)	0.51
**Efficacy (3-month CT response, mRECIST criteria)**			
CR	3/19 (15.8%)	4/18 (22.2%)	0.69
PR	9/19 (47.4%)	10/18 (55.6%)	0.75
SD	4/19 (21%)	2/18 (11.1%)	0.66
PD	3/19 (15.8%)	2/18 (11.1%)	1
ORR	12/19 (63.2%)	14/18 (77.8%)	0.48
DCR	16/19 (84.2%)	16/18 (88.9%)	1

TACE: transarterial chemoembolization; CTCAE: common terminology criteria for adverse events; CT: computed tomography; mRECIST: modified response evaluation criteria in solid tumors; CR: complete response; PR: partial response; SD: stable disease, PD: progression disease; ORR: overall response rate; DCR: disease control rate.

## Data Availability

The data presented in this study are available on request from the corresponding author due to privacy.
